# Multi‐Resistant *Staphylococcus aureus* Growth Inhibition Using an Innovative High Voltage Nanosecond Pulser: In Vitro Experimental Results

**DOI:** 10.1002/mbo3.70126

**Published:** 2025-12-09

**Authors:** Stavros Balasis, Konstantinos Papageorgiou, Sophia Georgiou, Fevronia Kolonitsiou, Nikolaos Giormezis, Antonios Kyriakopoulos, Chrysa Oikonomou, Georgios‐Filippos Papageorgiou

**Affiliations:** ^1^ Department of Orthopedics University of Patras, Medical School Patras Greece; ^2^ School of Engineering, Department of Financial and Management Engineering University of the Aegean Chios Greece; ^3^ Department of Dermatology University of Patras, Medical School Patras Greece; ^4^ Department of Microbiology University of Patras, Medical School Patras Greece; ^5^ Plastic Surgery Department Evaggelismos Hospital Athens Greece; ^6^ Faculty of Medicine University of Craiova Craiova Romania

**Keywords:** electric field pulser, electroporation, nanosecond, radiofrequency pulses, *staphylococcus aureus*

## Abstract

Multi‐Resistant Bacteria (MRB) is a threatening biomedical problem, whose solution is of paramount importance. Due to the antibiotics resistance there is an emerging need for novel treatment strategies and protocolls. As bacteria tolerance in modern chemotherapeytic agents expands, the introduction of alternative methods is fundamental. The use of High voltage Electric Pulses, through a process known as Irreversible Electroporation (IRE), is an effective alternative bacterial control method. This paper describes a new prototype high voltage nanosecond pulser and validates its effectiveness in the in‐vitro growth inhibition of a clinical resistant *Staphylococcus aureus* strain. Radiofrequency (RF) pulses of 100 ns and 450 ns pulse width and 1 Hz and 1 kHz repetition rate respectively were tested for therapy time in the range of 20–200 s. Increasing the electric field strength up to 11.5 kV/cm and the duration of therapy time up to 200 s results in 3.5 log scale reduction in bacterial cells. Nanosecond electric pulsed fields from our prototype device inhibite *S. aureus* growth in in‐vitro test. It is sugested to test our prototype device in ex‐vivo studies and propose a therapeutic protocol for infected skin wounds.

## Graphical abstract

In vitro *S. aureus* growth inhibition using RF radiation.

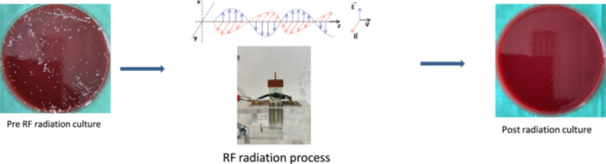

**Abbreviations**
CDC: Centers for Disease Control and Prevention
CFU/ml: Colony Forming Units per milliliter
HAI: Health Acquired Infections
MRB: Multi Resistant Bacteria
MRSA: Methicillin Resistant *Staphylococcus aureus*
nsPEF: nanosecond Electric Pulsed Fields
OECD: Organization for Economic Co‐operation and Development
PEFs: Electric pulsed fields
PRF: pulse repetition rate
RF: Radiogrequency
WHO: World Health Organization

## 1 Introduction

Multi‐drug resistant bacteria (MRB) are an emerging medical and public health problem worldwide (WHO 2021; CDC 2019; OECD 2022). Health care acquired infections (HAI's) increase morbidity and mortality and health care services expenses (WHO 2011). Trauma patients that require prolonged hospitalization (Eisner et al. 2020), severe burned patients (Leseva et al. 2013), and patients with chronic wounds and ulcers (Haesler et al. 2019) are in major risk for developing infections with MRB strains. Surgical debridement and sterile dressing changes with additional intravenous antibiotic therapy is the golden standard treatment (Thomas et al. 2021). Because of the antibiotic resistance, as well as their side effects and toxicity in patients, introduction of alternatives wound disinfection and treatment methods is fundamental.

Electric pulsed fields (PEFs) have been a well known treatment modality in liver and prostate cancer since 1990 (Breton and Mir 2012). High voltage electric fields can induce a process known as irreversible electroporation that promotes cancer cells death either directly, or via apoptotic pathways (Geboers et al. 2020). Electric pulsed fields have been used in food disinfection (Campbell et al. 2008; Garner 2019), in water treatment systems (Huo et al. 2022) and drug sterilization (Golberg et al. 2009). Therefore, pulsed electric fields devices and protocols for wound and implant disinfection, burnt area debridement (Wu et al. 2021) and osteomyelitis treatment have been introduced (Muñoz et al. 2019).

So far, pulsed electric field application on multidrug‐resistant bacteria has displayed very promising results in in‐vitro and in‐vivo studies. Technical difficulties such as electric field pulser characteristics on the one hand, design and construction for the delivering probe on the other hand are the major limitations of the method.

Most of existing electric fields pulsers are producing electric pulses of several kV/cm with a pulse width of 1μsec (Puc et al. 2004). In our study we try to examine the impact of applying electric pulsed fields with pulse width in the order of 50‐100 ns from our developed prototype electric field pulser. We studied Electric field strength, pulse width, repetition rate and treatment time and their impact in in‐vitro growth inhibition of a methicillin resistant clinical strain of *Staphylococcus aureus* (MRSA).

## 2 Materials and Methods

### 2.1 High Voltage Nanosecond Pulser Design and Building

**Figure 1:**
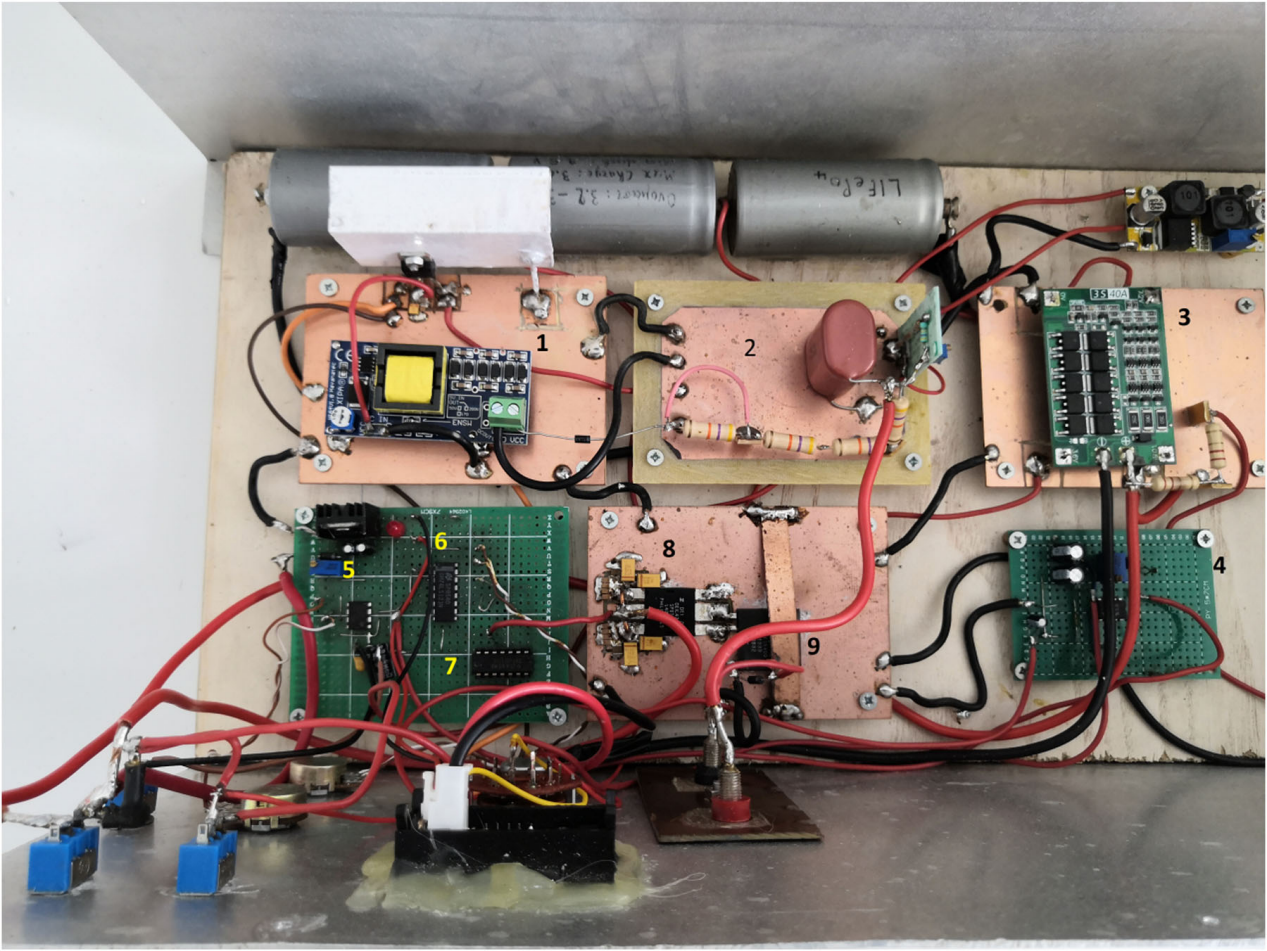
The prototype High Voltage nanosecond pulser. The labels from 1 to 9 indicate the HV generation circuit, the 1μF/2000V capacitor for the energy storage, the batteries regulation electronic circuit, the LM555 timer, the retriggerable monostate multivibrator, the D flip flop 74AC74, the MOSFET Driver DEIC420 and the extremely fast High Voltage power MOSFET IXTF1N400 by IXYS respectively.

**Figure 2:**
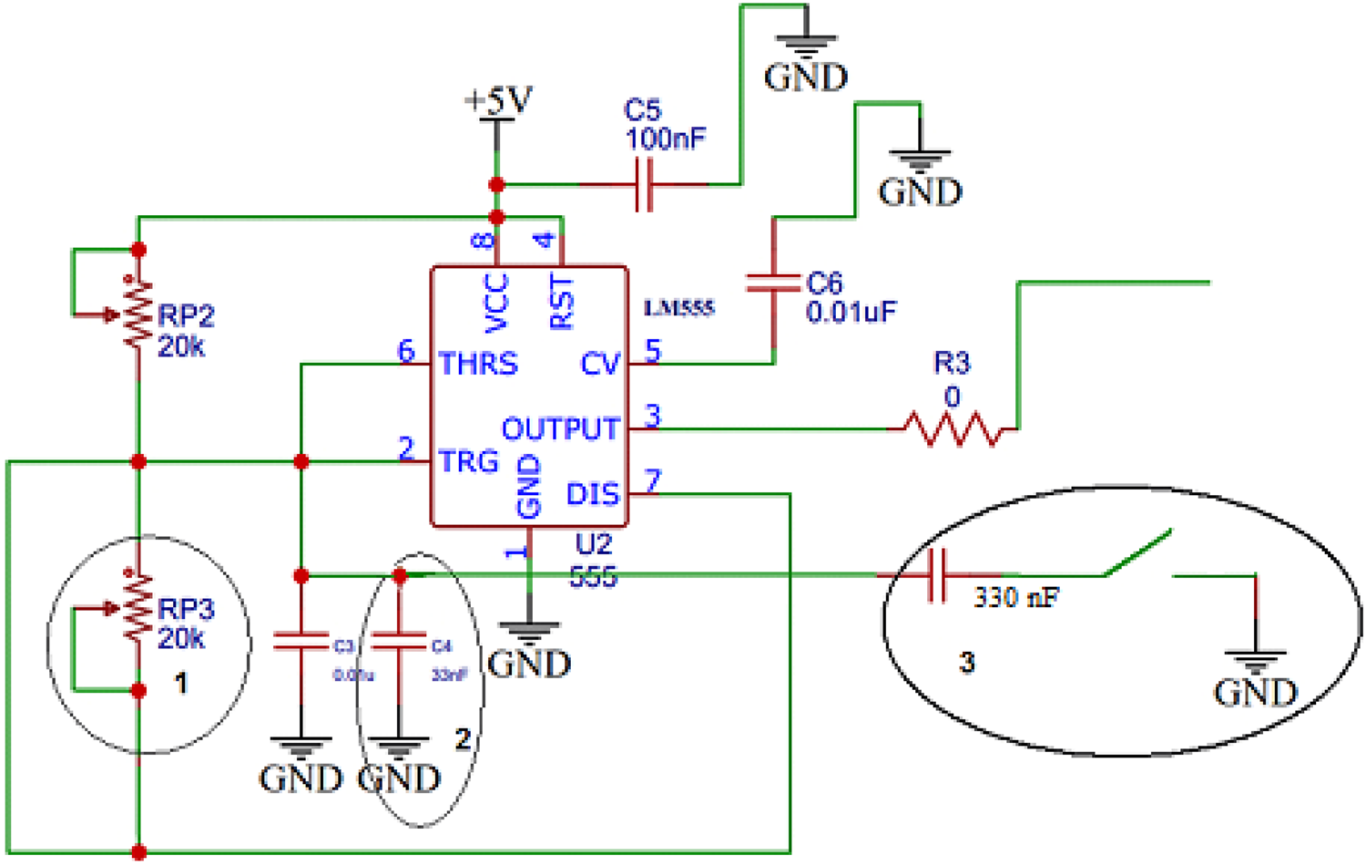
The electronic circuit of the LM555 timer.

To fulfill the main objectives of this study, a prototype custom made high voltage MOSFET‐based nanosecond pulser (Papageorgiou and Polychroni 2021) able to develop an adjustable high voltage up to 1200 VDC was used. The innovative nanopulser in Figure 1

it has been designed and constructed in the Research Physics Laboratory of the Aegean University and could operate using a 9.6VDC from 3 rechargeable batteries LiFeP_4_. The duration of the RF pulses is variable in the range between 50 ns and 500 ns and the pulse repetition rate (PRF) is also selectable and adjustable in two bands; one ranging from 1 to 10 Hz and the other from 1 to 146 kHz with the help of an external switch located in the front panel of the device. The switch is actually engaging an additional capacitor C_5_ of 330 nF connected in parallel with the other two existing capacitors C_3_ and C_4_ in the LM555 electronic circuit shown in Figure 2 and also connected with a variable resistance and a potentiometer allowing the selection of the desirable band of the pulse repetition rate.

The high voltage module is based on the integrated circuit HIA4V1.4 from Xtechnicis rated at 10 W. It converts the battery's 9.6VDC to a selectable high voltage output with the help of a potentiometer and stores this charge in a 1μF/2000Vcapacitor and deliver it back to the circuit to supply power to the probe when the pulse is generated.

Α general‐purpose timing integrated circuit, LM555, was used as a clock, where in the mode of unstable operation it generates a TTL square pulse with a frequency ranging from 1 kHz up to 146 kHz. To achieve the unstable operation of the circuit, two variable resistors of 20kΩ each have been placed according to the manufacturer's instructions (Fairchild Semiconductors 2002). One of those resistors is placed on the panel of the device and allows the user to change the repetition time of the pulse.

The output signal from the LM555 clock circuit is then splitted and connected in a dual integrated circuit consisting of two identical retriggerable monostable multivibrators (74LS123). The characteristics of the pulses produced by the 74LS123 integrated circuit are determined from the values of an external resistor and a capacitor with values of 8.2KΩ and 33 pF respectively, to create a strong pulse of short duration according to the design specifications, since 5kΩ is the manufacturer's minimum value and the smaller the resistance value the smaller the pulse width.

Also, a variable resistance of 20kΩ in series with a potentiometer located in the front panel of the device has been connected to the external resistance of the one multivibrator circuit to differentiate the two pulses produced and adjust the pulse duration. Therefore, based on the values of the capacitors and resistors chosen for this specific application, the pulse duration theoretically is ranging from 50 ns to 330 ns. The two pulses generated at the outputs of the 74LS123 monostable multivibrator, are used to drive the inputs of the 74AC74 D flip‐flop, the output of which corresponds to the time difference of the two pulses and represents the main pulse used to feed the MOSFET driver. It is worth noting that a 10nF SMD capacitor was placed on the supply terminal of the D flip flop 74AC74 to reduce spurious noise and there is also a + 5 V supply junction from the LM7805 voltage regulator to both the 74AC74 and 74LS123 integrated circuits.

A high speed high current Transistor–Transistor Logic (TTL‐CMOS) MOSFET driver DEIC420 by IXYS was used to bias the MOSFET at extremely short rates. The CMOS/TTL input of the driver is connected to the output of the pulse generation circuit. The driver is required because of the extremely high current necessary to charge the MOSFET gate capacitance. The MOSFET driver is powered using the battery terminals directly so that the maximum current is available for the gate charging and discharging. Three different capacitors, ranging in capacitance, are used to stabilize and filter the power supply to the MOSFET driver at the time of demand. The different capacitance ranges are used to cover different ranges of high frequency noises. More specifically, 4 tantalum capacitors of 4.7 µF, 8 ceramic capacitors of 0.01 µF and 4 ceramic capacitors of 0.3 µF were used. Important parameters such as the minimum possible copper track distances for the signal input, fully balanced current supply, decoupling capacitors of the MOSFET driver, driver ground as well as the use of capacitors of different order of capacitance range for decoupling oscillations as well as their good quality were taken into account in the construction of the board.

The design specifications require pulse durations ranging between (50‐300)ns, so an ultra‐fast RF MOSFET (IXTF1N400) RF Power MOSFET‐ N type) from IXYS, rated at 4000 V was selected as the switch. The N‐channel MOSFET switch is a type of semiconductor categorized under the FET (Field Effect Semiconductor) category. The operation of the MOSFET semiconductor relies on the gate capacitor being charged or discharged to open or close the gate of the switch.

The main reasons that the specific MOSFET switch IXTF1N400 was chosen is the high operating frequency, the low Turn On Delay time (Td On)and low Turn Off Delay time (Td Off) as well as that it provides infinite resistance and endurance up to 1200 V which depends from the gate electrostatic field alone. The source terminal of the MOSFET is connected to the ground and the drain is connected to the high voltage, through two resistors whose purpose is to limit the current, not to exceed 8 A if for some reason the switch is kept closed. A fast switching diode is placed inversely to protect the MOSFET from reverse voltages and inductive loads. Inside the IXTF1N400 MOSFET component the manufacturer has placed another similar diode for the same reason.

**Figure 3:**
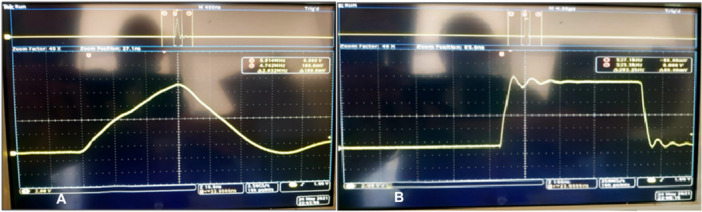
(A) Pulse output of the MOSFET IXTF1N400 in the case of minimum operating parameters. The pulse shown has a height of 100 V (2 V/divx 20 probe) and a period of approximately 60 ns (B) Pulse output of the MOSFET IXTF1N400 in the case of maximum operating parameters. The pulse shown has a height of 100 V (2 V/divx 20 probe) and a period of 400 ns.

Figure 3 shows the pulse output of the MOSFETIXTF1N400 in the case of (a) minimum and (b) maximum operating parameters).

### 2.2 Electrporation Cuvette

We used 0.1 cm gap Electroporation cuvettes (Gene Pulser/MicroPulser Electroporation Cuvettes, 0.1 cm gap, Bio‐rad Co).

### 2.3 Bacteria Strain

We added 70 μl of MRSA suspension with a final concentration 10^6^ CFU/ml in each electroporation cuvette (Gene Pulser/MicroPulser Electroporation Cuvettes, 0.1 cm gap, Bio‐rad Co) and applied nanosecond pulsed electric field (nsPEF) using two treatment protocols: a) 100 ns pulse width, 1 kHz repetition rate b) 450 ns pulse width, 1 Hz repetition rate. For each of the above conditions we treat bacteria with the RF nanosecond pulser for 20, 50, 80, 100, 120, 150 and 200 s respectively. Immediately after nsPEF treatment, 1 μl of each radiated electroporation cuvette solution and 1 μl of our control irradiated cuvette solution was cultured in blood agar at 37°C for 24 h. After overnight culture we counted the remaining bacterial CFU/ml (colony‐forming units per milliliter) after nsPEF treatment and the bacterial CFU/ml concentration of the control cuvette solution respectively. We repeated the experiment three times for each of the above conditions.

Data and materials are available on: https://osf.io/n9v2j/.

### 2.4 Statistical Analysis

SPSS 28.0 (IBM SPSS Statistics for Windows, Version 28.0. Armonk, NY: IBM Corp) was used for statistical analysis utilizing two‐tail Pearson correlation test for depended variables. *P* values of less than 0.05 considered as significant.

## 3 Results

**Figure 4:**
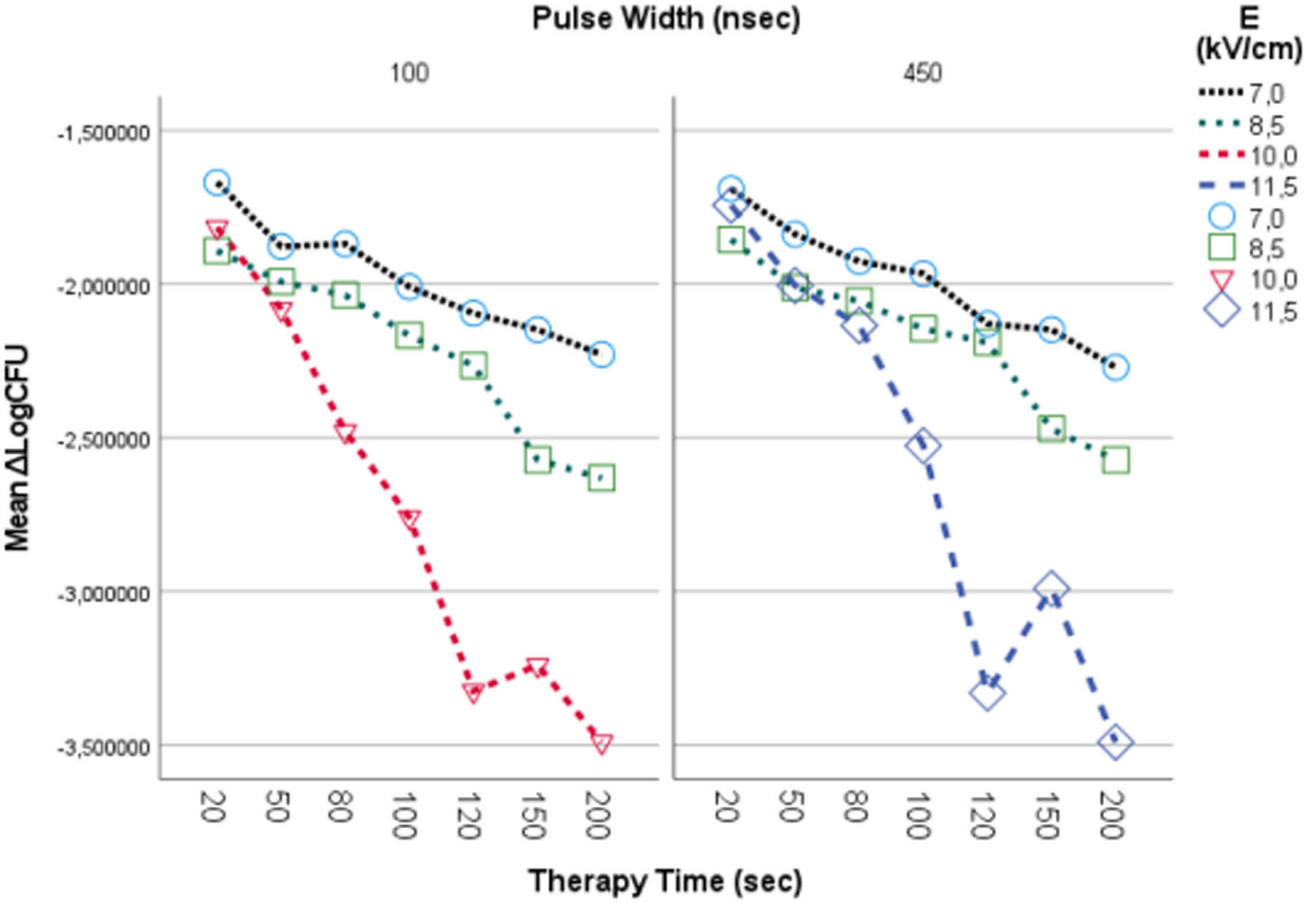
Y axis represents Mean ΔLogCFU (CFUtest‐CFUcontrol). X axis represents therapy time in seconds. Black dotted line represents 7 kV/cm, green dotted line represents 8.5 kV/cm, red line represents 10 kV/cm and blue line represents 11.5 kV/cm. In left column are represented results for 100nsec pulse width and 1 kHz repetition rate. In right column are represented results for 450 nsec pulse width and 1 Hz repetition rate.

After an overnight incubation we count the surviving bacteria cultured in blood agar. The results are given in Figure 4

Increasing both the electric field strength and therapy time resulted in an increase of bacteria growth inhibition up to 3.49 in logarithmic scale. This inhibition is strongly correlated to electric field strength and treatment time (*p* < 0.01) as shown in Table 1.

**Table 1:** Correlations between Electric field strength, pulse width, repetition rate, therapy time and inhibition in bacteria cells.

Correlations		E (kV/cm)	Pulse Width (nsec)	Repetition Rate (Hz)	Therapy Time (sec)	ΔLogCFU
E (kV/cm)	Pearson correlation	1	0.104	−0.104	0.021	−0.438**
	Sig. (2‐tailed)		0.191	0.191	0.795	0.000
	N	161	161	161	161	161
Pulse width (nsec)	Pearson correlation	0.104	1	−1.000**	−0.026	0.122
	Sig. (2‐tailed)	0.191		0.000	0.747	0.124
	N	161	161	161	161	161
Repetition rate (Hz)	Pearson correlation	−0.104	–1.000**	1	0.026	−0.122
	Sig. (2‐tailed)	0.191	0.000		0.747	0.124
	N	161	161	161	161	161
Therapy time (sec)	Pearson correlation	0.021	−0.026	0.26	1	−0.596**
	Sig. (2‐tailed)	0.795	0.747	0.747		0.000
	N	161	161	161	161	161
ΔLogCFU	Pearson correlation	−0.438**	0.122	−0.122	−0.596**	1
	Sig. (2‐tailed)	0.000	0.124	0.124	0.000	
	N	161	161	161	161	161

** Correlation is significant at the 0.01 level (2‐tailed).

**Figure 5:**
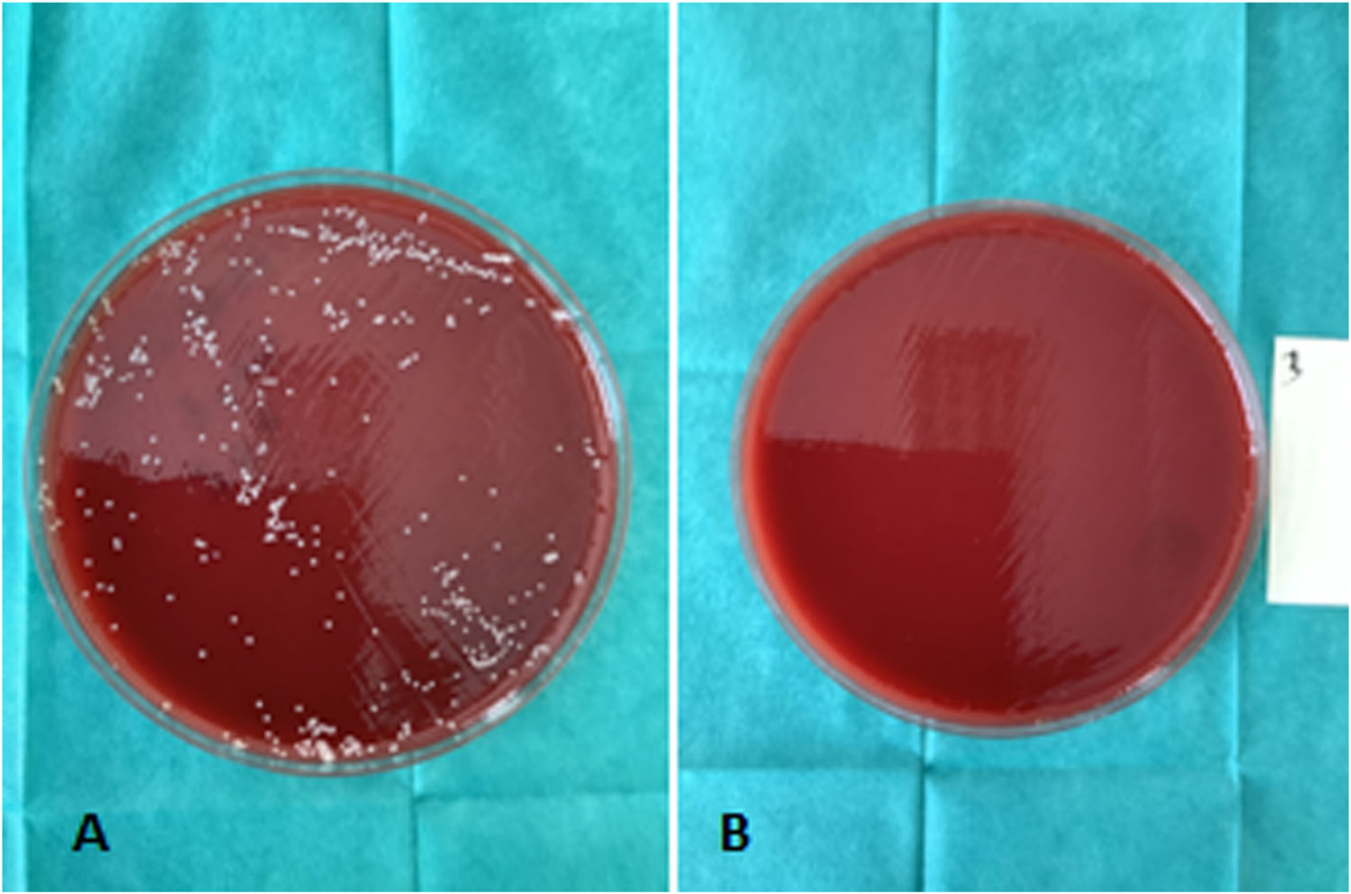
(A) *S. aureus* CFU after overnight incubation without treatment. (B) *S. aureus* CFU after nsPEFs treatment.

The best results were observed for treatment time 120 and 200 s for both E = 10 kV/cm, 100nsec pulse width, 1 kHz repetition rate frequency and E = 11.5 kV/cm, 450 nsec pulse width, 1 Hz repetition rate frequency (0–1 count CFU on Petri dishes or 5 log scale reduction) Figure 5

**Figure 6:**
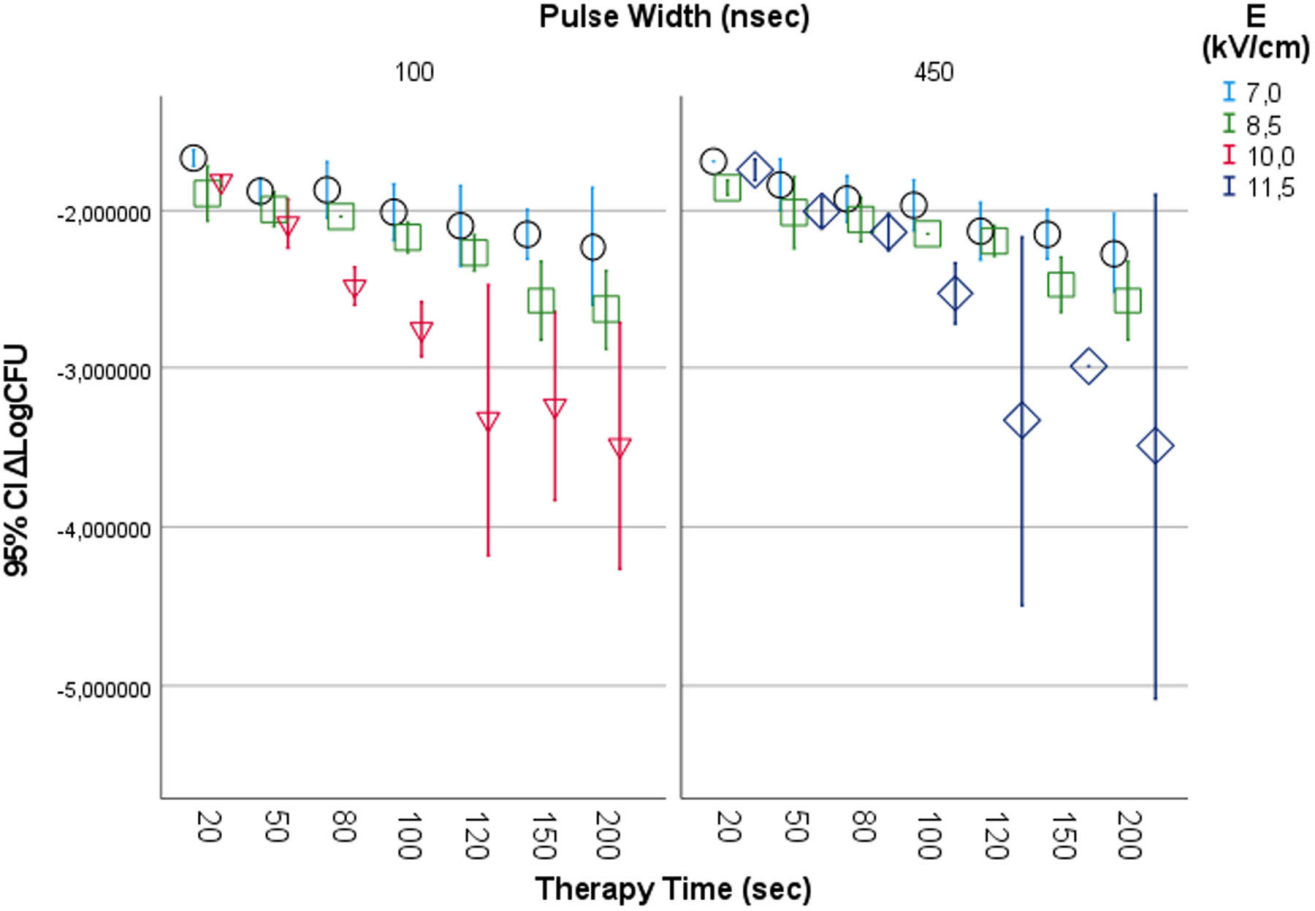
In Y axis we represent ΔLogCFU, in X axis we represent therapy time in sec. Light blue circle represents 7 kV/cm electric field, green square represents 8.5 kV/cm electric field, red triangle represents 10 kV/cm electric field and deep blue rhombus represents 11.5 kV/cm electric field for 100 ns pulse width (left column) and for 450 ns pulse width (right column).

Increasing the electric field strength increases bacterial growth inhibition for each treatment time Figure 6.

**Figure 7:**
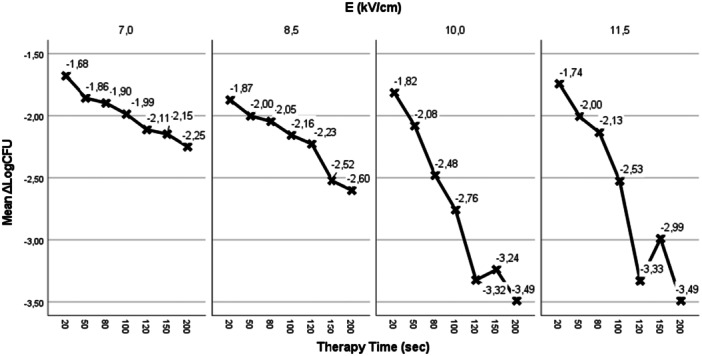
Increased bacterial inhibition increasing electric field strength and therapy time.

Applying PEFs on electroporation cuvettes initially of Electric Field Strength of 7 kV/cm and assessing their effect on CFU reduction, after offering 20, 50, 80, 100, 120, 150 and 200 s treatment time using pulses 100 nsec pulse width, 1 kHz repetition rate frequency and 450 nsec pulse width, 1 Hz repetition rate frequency resulted in1.68‐2.25 Log scale reduction (LogCFU/ml_control_‐LogCFU/ml_test_) in bacterial culture Figure 7.

Increasing the electric field strength up to 8.5 kV/cm for both the experiment setting conditions (100 nsec pulse width, 1 kHz repetition rate frequency and 450 nsec pulse width, 1 Hz repetition rate frequency) for 20, 50, 80, 100, 120, 150 and 200 s treatment time results in an increased bacterial reduction 1.87−2.6 Log scale Figure 7. The best result was for treatment time 200 s using 450 nsec, 1 Hz pulses.

Higher electric field strength of 10 kV/cm, at 100 nsec pulse width, 1 kHz repetition rate frequency, had an increased effect in bacterial reduction. The best result was for 120 and 200 s that showed a 3.5 scale log reduction (3.32−3.49) Figure 7. Using the above parameters resulted in negative post treatment cultures (CFU_test_ = 0) Figure 5.

Using our electroporator's strongest electric field of 11.5 kV/cm, at 450nsec pulse with and 1 Hz repetition rate frequency resulted in a 3.5 log scale reduction in bacteria (3.33–3.49) Figure 6 and Figure 7. The best results were for 120 and 200 s treatment time.

## 4 Discussion

**Figure 8:**
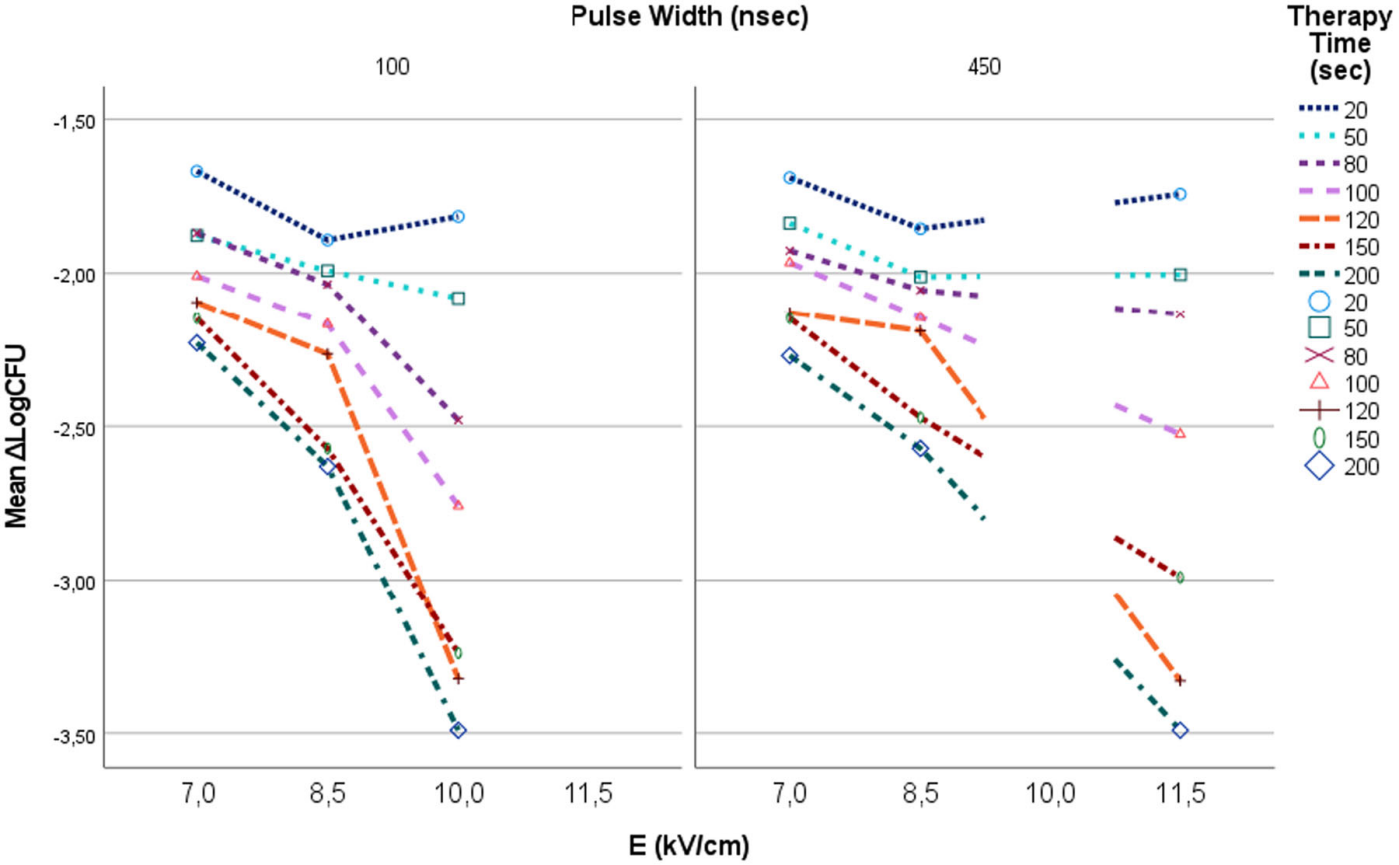
Increasing bacterial inhibition as therapy time increases.

We test bacterial growth inhibition using low repetition rate of the pulsed field, 1 Hz, and high repetition rate pulsed fields, 1 kHz. There is strong correlation between treatment time, electric field strength and bacteria cell growth inhibition. Increasing the treatment time enhances the bacteria growth inhibition Figure 7 and Figure 8.

In short treatment time (< 80 s) there is a 1.5−2.48 Log scale reduction in bacteria cell. Increasing treatment time till 200 s increases bacterial growth inhibition. For treatment time 80−200 s there is a 1.99−3.49 log reduction in bacteria cells Figure 8. The best result was for 450 ns pulse width, 11.5 kV/cm electric field, 1 Hz repetition frequency and 200 s treatment time.

Pulse width and repetition rate frequency have not statistical significant correlation with bacteria cell inhibition (*p* = 0.161), even though 450 nsec pulses were slightly more effective than 100 nsec pulses at the same electric field strength.

Total energy transferred did not overcome 10^−3 ^J and there was not temperature increase over 0.778°C in cuvette solution. Short time electric field pulses through irreversible electroporation lead bacterial cells to death and they cannot repair cell membrane damage even though we reculture them in best conditions. As we previous mentioned it is the electric field strength that destroys bacterial cell membrane and not the pulse width. We used very short pulse width pulses to investigate their efficacy taking into consideration that long pulses may affect cardiac rhythm. Taking into consideration that 1 Hz repetition rate protocol transferred 1000times less energy while the results were similar we suggest that it is the electric field strength pulsed in ns that affect in bacterial cell viability and not the energy transferred.

## 5 Conclusions – Future Plans

We found increasing bacterial growth inhibition using nsPEFs from our prototype High voltage nanosecond electric field pulser corresponding to increasing of the electric field strength. We tested on low 1 Hz and high 1 kHz repetition rate frequency and found no significant differences in inhibiting bacterial growth, while at the same time the effectiveness of our method increases rapidly until 120 s therapy time and continues up to 3,5 log at 200 s. After those promising initial results, the in‐vitro effectiveness of our electric field pulser in medium (5, 10 Hz) and very high (2500 Hz) repetition rate frequencies is going to be examined. Additionally, assessing our prototype pulser efficacy in growth inhibition of referencing strains both Gram positive and Gram negative bacteria and eukaryotic cells (e.g. yeasts) is becoming a promising field we are going to perform. In vitro studies on fibroblast survival in cell cultures after nsPEFs will allow us to test the method in live models. Also in our future plans is to apply nsPEFs through slim interdigital electrodes with best adaptation to infected ulcer area.

## Author Contributions

**Stavros Balasis:** conceptualization, methodology, investigation, writing – review and editing, writing – original draft, formal analysis, software, data curation, validation, resources. **Konstantinos Papageorgiou:** conceptualization, methodology, funding acquisition, supervision, writing – original draft, writing – review and editing, project administration, data curation, validation, investigation, resources. **Sophia Georgiou:** project administration, funding acquisition, writing – original draft, writing – review and editing, data curation, conceptualization, methodology, validation, investigation, supervision, resources. **Fevronia Kolonitsiou:** project administration, supervision, writing – original draft, writing – review and editing, methodology, validation, data curation. **Nikolaos Giormezis:** writing – review and editing, methodology, validation, data curation. **Antonios Kyriakopoulos:** writing – review and editing, writing – original draft, software. **Chrysa Oikonomou:** writing – original draft, writing – review and editing, visualization. **Georgios‐Filippos Papageorgiou:** writing – original draft, writing – review and editing, software, resources, investigation.

## Ethics Statement

The authors have nothing to report.

## Consent

The authors have nothing to report.

## Conflicts of Interest

The authors declare no conflicts of interest.

## Data Availability

The data that support the findings of this study are openly available in https://osf.io/sx9k5 on https://osf.io/n9v2j/.
